# JC Virus-DNA Detection Is Associated with CD8 Effector Accumulation in Peripheral Blood of Patients with Multiple Sclerosis under Natalizumab Treatment, Independently from JC Virus Serostatus

**DOI:** 10.1155/2018/5297980

**Published:** 2018-02-27

**Authors:** Maria A. Zingaropoli, Marco Iannetta, Simona Pontecorvo, Elena Anzivino, Carla Prezioso, Donatella Maria Rodio, Manuela Morreale, Alessandra D'Abramo, Alessandra Oliva, Miriam Lichtner, Antonio Cortese, Marco Frontoni, Valeria Pietropaolo, Ada Francia, Claudio M. Mastroianni, Vincenzo Vullo, Maria R. Ciardi

**Affiliations:** ^1^Department of Public Health and Infectious Diseases, Sapienza University, Rome, Italy; ^2^National Institute for Infectious Diseases Lazzaro Spallanzani, IRCCS, Rome, Italy; ^3^Department of Neurology and Psychiatry, Multiple Sclerosis Center, Sapienza University, Rome, Italy; ^4^Department of Medical and Surgical Sciences and Biotechnology, Neurovascular Diagnosis Unit, Section of Neurology, Sapienza University, Rome, Italy

## Abstract

Although natalizumab (anti-*α*4 integrin) represents an effective therapy for relapsing remitting multiple sclerosis (RRMS), it is associated with an increased risk of developing progressive multifocal leukoencephalopathy (PML), caused by the polyomavirus JC (JCV). The aim of this study was to explore natalizumab-induced phenotypic changes in peripheral blood T-lymphocytes and their relationship with JCV reactivation. Forty-four patients affected by RRMS were enrolled. Blood and urine samples were classified according to natalizumab infusion number: 0 (*N*0), 1–12 (*N*12), 13–24 (*N*24), 25–36 (*N*36), and over 36 (*N* > 36) infusions. JCV-DNA was detected in plasma and urine. T-lymphocyte phenotype was evaluated with flow cytometry. JCV serostatus was assessed. Ten healthy donors (HD), whose ages and sexes matched with the RRMS patients of the *N*0 group, were enrolled. CD8 effector (CD8 E) percentages were increased in natalizumab treated patients with detectable JCV-DNA in plasma or urine compared to JCV-DNA negative patients (JCV−) (*p* < 0.01 and *p* < 0.001, resp.). Patients with CD8 E percentages above 10.4% tended to show detectable JCV-DNA in plasma and/or urine (ROC curve *p* = 0.001). The CD8 E was increased when JCV-DNA was detectable in plasma or urine, independently from JCV serology, for *N*12 and *N*24 groups (*p* < 0.01). As long as PML can affect RRMS patients under natalizumab treatment with a negative JCV serology, the assessment of CD8 E could help in the evaluation of JCV reactivation.

## 1. Introduction

More than 2.3 million people worldwide are living with multiple sclerosis (MS) [1], a chronic inflammatory demyelinating disease of the central nervous system (CNS) [2] with 85–90% of cases presenting as a relapsing remitting (RR) form [1]. Lymphocyte migration through the blood brain barrier (BBB) represents a crucial step in the process that leads to inflammation and demyelination in the CNS [3]. The adhesion and subsequent migration of leukocytes into the CNS are regulated by the molecular interaction between membrane receptors and their cognate ligands, which are expressed on endothelial cell surface. Specifically, lymphocyte migration into the CNS is regulated by the interaction of the *α*4*β*1 integrin (very late antigen [VLA]-4), expressed on their surface, with the vascular-cell adhesion molecule (VCAM)-1, expressed on the vascular endothelial cell surface of CNS blood vessels [4, 5]. Natalizumab (Tysabri; Biogen Idec, Cambridge, MA, USA), a humanized monoclonal antibody targeting the *α*4 integrin (CD49d), was developed to inhibit leukocyte adhesion to the vascular endothelium of the CNS [4, 6]. Consequently, natalizumab limits leukocyte recruitment in the CNS, reducing the inflammation responsible for MS lesions [7].

Natalizumab is a highly effective therapy for RRMS characterized by a significant reduction in the relapse rate and risk of disability progression [8, 9]. However, this treatment is associated with an increased risk of progressive multifocal leukoencephalopathy (PML), caused by the John Cunningham polyomavirus (JCV) reactivation. Although natalizumab was initially approved for RRMS by the US Food and Drug Administration (FDA) in 2004 [10, 11], in 2005 it was temporarily withdrawn from the market because of the occurrence of three cases of PML (two patients with MS and one with Crohn's disease) [12–14].

Factors associated with increased risk of developing PML are (1) long-term natalizumab treatment (beyond 24 months), (2) prior immunosuppressive treatment for MS, and (3) JCV positive serology (assessed with the Stratify JCV® test) [15]. However, the presence of anti-JCV antibodies was neither diagnostic nor prognostic for PML, considering that patients with negative serology for JCV can develop PML [16].

Several studies showed that natalizumab treatment affects lymphocyte circulating subsets, increasing the pool of T-cells and other lymphocytes in peripheral blood [17–19] and modifying T-cell balance with an increased activation state and potential to produce proinflammatory cytokines [17, 20, 21]. A few studies have addressed the issue of immunologic changes related to JCV reactivation and PML onset [17, 22, 23].

The aim of this study was to describe natalizumab-induced phenotypic changes in peripheral blood T-lymphocytes and their relationship with JCV reactivation, with particular interest for JCV seronegative subjects.

## 2. Materials and Methods

### 2.1. Study Population

Patients affected by RRMS, followed up at the Department of Neurology and Psychiatry of the University of Rome “Sapienza,” were enrolled from March 2013 to March 2016. All patients were under a natalizumab-based treatment and regularly underwent a complete physical and neurological examination. Neurological disability was assessed with the Expanded Disability Status Scale (EDSS) score.

This study was approved by the Ethics Committee of Policlinico Umberto I of Rome (protocol number 130/13). All participants fulfilled the Italian Agency of Drug (AIFA) criteria for natalizumab treatment and provided a written informed consent before enrollment.

### 2.2. Sample Collection

Urine and blood were collected from RRMS patients before natalizumab administration. Samples were grouped according to the number of natalizumab infusions, in the following groups: before starting the treatment (*N*0), from 1 to 12 infusions (*N*12), from 13 to 24 infusions (*N*24), from 25 to 36 infusions (*N*36), and over 36 infusions (*N* > 36). Ten healthy donors (HD), whose ages and sexes matched with the RRMS patients of the *N*0 group, were enrolled as control group.

Whole blood was collected in ethylenediaminetetraacetic acid (EDTA) and heparin tubes. Serum was obtained from blood collected in tubes without anticoagulants, after centrifugation, and stored at −80°C. Plasma and peripheral blood mononuclear cells (PBMC) were isolated from EDTA whole blood via Ficoll Histopaque (Amersham Biosciences, Uppsala, Sweden) density gradient centrifugation. The number of viable leukocytes was determined by trypan blue exclusion. Plasma and PBMC were stored at −80°C. Urine was collected in sterile screw-cap containers and stored at −80°C. Heparin whole blood was used for multicolor flow cytometry immunophenotyping.

### 2.3. Serological and Virological Analysis

The presence of JCV specific antibodies in the serum of RRMS patients was assessed after the activation of the Stratify JCV service, supported by Biogen Idec, using the Stratify JCV assay, a two-step enzyme-linked immunosorbent assay, able to detect anti-JCV specific IgG. Both first-generation (Stratify JCV assay, Focus Diagnostics, Cypress, CA) and second-generation (Stratify JCV DxSelect, Focus Diagnostics, Cypress, CA) assays were used to detect anti-JCV antibodies in this cohort of patients, according to enrollment date and test availability. Viral DNA was extracted from plasma and urine samples with the DNeasy Blood & Tissue Kit (QIAGEN, S.p.A, Milan, Italy), according to the manufacturer's instructions, and the extraction product was analyzed with a real time PCR (qPCR) system to detect and quantify a 54-bp amplicon in the JCV T antigen region using a 7300 real time PCR system (Applied Biosystems, USA). The viral load results have been reported as the mean of two positive reactions for each sample, and the JCV-DNA load in plasma and urine specimens has been expressed as genome equivalents (gEq)/milliliter of sample. Negative and positive controls have been included in each qPCR session. Standard curve was obtained from serial dilutions (range: 10^5^–10^2^ gEq/mL) of a plasmid containing the entire JCV genome [24]. The lower detection limit of the qPCR system was 10 DNA copies of the target gene per amplification reaction, corresponding to 10 genome equivalents per reaction (10 gEq/reaction).

JCV-DNA positive samples were further analyzed using a nested PCR system for the amplification of the hypervariable noncoding control region (NCCR) in order to confirm the positivity obtained with the real time system, thus increasing specificity [24].

### 2.4. T-Lymphocyte Analysis

Immunofluorescence stainings for CD4+ and CD8+ T-lymphocyte subsets, immune activation and senescence, were performed according to a lyse-and-wash protocol using heparin whole blood samples. Briefly, T-cell immune activation was assessed measuring the percentages of double positive HLA-DR and CD38 cells, while immune senescence was assessed measuring the percentages of CD28 negative and CD57 positive cells for both CD3+CD4+ and CD3+CD8+ T-lymphocytes. In order to evaluate T-lymphocyte subsets, CD4 and CD8 subpopulations were defined according to CD27 and CD45RO expressions, as follows: naïve (N: CD27+CD45RO−), central memory (CM: CD27+CD45RO+), effector memory (EM: CD27−CD45RO+), effector (E: CD27−CD45RO−), and, only for CD8, intermediate (I: CD27lowCD45RO−). Flow cytometry staining protocols and analyses have been thoroughly described in detail elsewhere [17].

### 2.5. Data Analysis and Statistics

Flow cytometry data were analyzed using FlowJo Software v. 10. Statistical analyses were performed using GraphPad Prism version 6. The 2-tailed *χ*^2^ test or Fisher's exact test was used for comparing proportions. The Mann–Whitney and Kruskal–Wallis tests were used for comparing medians. Differences were considered significant if *p* ≤ 0.05.

## 3. Results

### 3.1. Study Population

Forty-four patients (24 females, 20 males) with clinically defined RRMS were included in the study. Median age [interquartile range (IQR)] was 38 [32.5–44.5]. Median [IQR] years of disease were 9 [7–14]. Before starting natalizumab, 61.4% of patients (27/44) had been treated with interferon *β*, 11.4% (5/44) with glatiramer acetate, and 4.5% (2/44) with mitoxantrone and interferon *β*, and 22.7% (10/44) had not received any previous treatment. The median [IQR] EDSS value was 2 [1-2]. Ten HD (5 females, 5 males), whose ages and sexes matched with RRMS patients of the *N*0 group, were enrolled as control group.

A total of 262 samples were collected from 44 RRMS patients (131 urine and 131 blood samples). Demographic and clinical features of all RRMS patients are shown in Table 1.

### 3.2. Comparison between JCV-DNA Detection and Stratify JCV Assay Results

Positivity rate of anti-JCV antibody detection was 42.1% (8/19), 30% (15/50), 40.5% (17/42), 50% (7/14), and 83.3% (5/6) for *N*0, *N*12, *N*24, *N*36, and *N* > 36, respectively (Supplementary Table 1). No statistically significant differences were found comparing the 5 groups of RRMS patients (2-tailed *χ*^2^-square test).

JCV-DNA was detected with qPCR in urine and plasma. JCV-DNA positivity rate in urine was 15.8% (3/19), 20% (10/50), 21.4% (9/42), 14.3% (2/14), and 16.7% (1/6), while in plasma it was 26.3% (5/19), 16% (8/50), 26.2% (11/42), 28.6% (4/14), and 16.7% (1/6) of cases for *N*0, *N*12, *N*24, *N*36, and *N* > 36, respectively. Combining plasma and urine JCV-DNA detection results, the positivity rate was 42.1% (8/19), 24% (12/50), 33.3% (14/42), 42.8% (6/14), and 33.3% (2/6) of cases for *N*0, *N*12, *N*24, *N*36, and *N* > 36, respectively. No statistically significant differences were found comparing the 5 groups in the three settings (urine, blood, or urine and blood) (2-tailed *χ*^2^ test).

At each sampling time, JCV serology and JCV-DNA detection in plasma and urine were compared. If JCV-DNA was detected either in plasma, urine, or both samples the patient was defined as JCV-DNA+ for that specific time point. Conversely, the patient was defined as JCV-DNA− if JCV-DNA was undetectable in all the samples analyzed for a specific time point.

**Figure 1:**
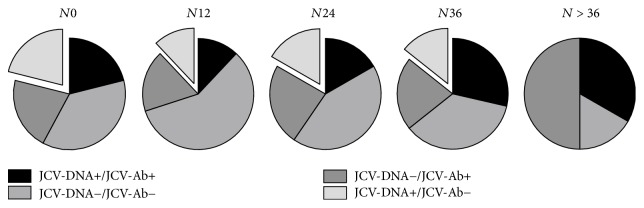
*Discordance between the Stratify JCV assay and JCV-DNA detection in blood and urine samples*. The pie charts represent the percentages of concordance and discordance between JCV antibody (Ab) and JCV-DNA detection in urine and plasma. Percentages of double positive JCV-DNA+ and JCV-Ab+ cases: 21% (4/19), 12% (6/50), 16.7% (7/42), 28.6% (4/14), and 33.3% (2/6) at *N*0, *N*12, *N*24, *N*36, and *N* > 36, respectively. Percentages of JCV-DNA− and JCV-Ab+ cases: 21% (4/19), 18% (9/50), 23,8% (10/42), 21.4% (3/14), and 50% (3/6) of cases at *N*0, *N*12, *N*24, *N*36, and *N* > 36, respectively. Percentages of cases with double negative JCV-DNA− and JCV-Ab−: 36,8% (7/19), 58% (29/50), 42,9% (18/42), 35.7% (5/14), and 16.7% (1/6) of cases at *N*0, *N*12, *N*24, *N*36, and *N* > 36, respectively. Percentages of discordant cases with JCV-DNA+ and JCV-Ab− (separated sector): 21.1% (4/19), 12% (6/50), 16.7% (7/42), 14.3% (2/14), and 0% (0/6) of cases at *N*0, *N*12, *N*24, *N*36, and *N* > 36, respectively. *N*0: 0 infusions, *N*12: from 1 to 12 infusions, *N*24: from 13 to 24 infusions, *N*36: from 25 to 36 infusions, and *N* > 36: over 36 infusions of natalizumab.

Comparing JCV serostatus and JCV-DNA detection in the 5 groups, a subset of patients with discordant results (JCV-DNA detection with a negative JCV serostatus) was identified: 21.1% (4/19), 12% (6/50), 16.7% (7/42), 14.3% (2/14), and 0% (0/6) of cases for *N*0, *N*12, *N*24, *N*36, and *N* > 36 groups, respectively. This subset of patients showed an active replication of JCV in either plasma, urine, or both samples, despite a negative serology for anti-JCV antibodies (Figure 1).

### 3.3. T-Lymphocyte Immunophenotyping

**Figure 2:**
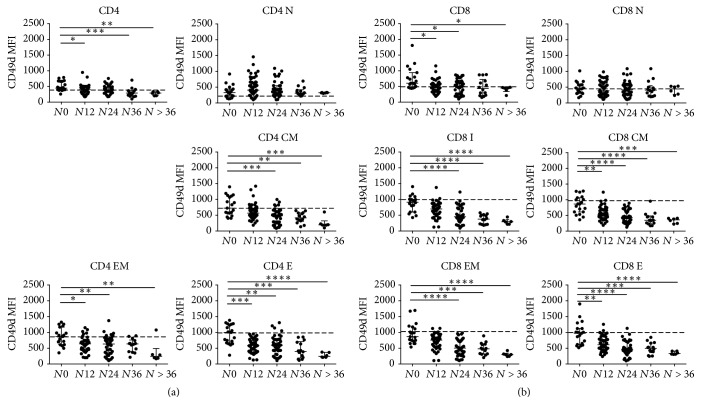
*CD49d median fluorescence intensity (MFI) measured on CD4+ and CD8+ T-lymphocyte subsets*. CD49d expression assessed on the overall CD4+ (a) and CD8+ (b) T-lymphocytes and their subpopulations in peripheral blood of RRMS patients under natalizumab treatment are represented after stratification according to natalizumab infusion number. Statistical analysis was performed using a one-way ANOVA test for nonparametrical data (Kruskal–Wallis): for CD4, *p* = 0.0004; CD4 N, *p* > 0,05; CD4 CM, *p* < 0,0001; CD4 EM, *p* = 0.0022; CD4 E, *p* < 0.0001; for CD8, *p* = 0.01; CD8 I, *p* < 0.0001; CD8 N, *p* > 0.05; CD8 CM, *p* < 0.0001; CD8 EM, *p* < 0.0001; CD8 E, *p* < 0.0001. Dunnett's posttest was performed comparing each group with *N*0, and asterisks represent the level of statistical significance. Dashed line represents median value of CD49d expression observed in healthy donors. CD49d expression on CD4+ and CD8+ T-lymphocytes decreased from *N*0 to *N* > 36 group with a linear trend (posttest for linear trend: *p* < 0,0001 and *p* = 0,0035, resp.). Lines and whiskers represent median values and interquartile ranges, respectively. N: naïve, CM: central memory, EM: effector memory, E: effectors, I: intermediate. *N*0: 0 infusions, *N*12: from 1 to 12 infusions, *N*24: from 13 to 24 infusions, *N*36: from 25 to 36 infusions, and *N* > 36: over 36 infusions of natalizumab. ^*∗*^0.05 < *p* < 0.01; ^*∗∗*^0.01 < *p* < 0.001; ^*∗∗∗*^*p* < 0.001; ^*∗∗∗∗*^*p* < 0.0001.

CD49d (*α*4 integrin) expression on CD4+ and CD8+ T-lymphocyte subsets was evaluated by measuring the median fluorescence intensity (MFI) with flow cytometry. CD49d expression on the overall CD4+ and CD8+ T-lymphocytes was decreased in patients with longer exposition compared to patients with shorter or no exposition to natalizumab treatment (Kruskal–Wallis test, *p* = 0.0004  and *p* = 0.01, resp.). Specifically, CD49d MFI remained unchanged on CD4 and CD8 *N* subsets in all the groups, while it was found decreased on CD4 and CD8 CM, EM, E, and CD8 I subsets of patients with longer exposition compared to patients with shorter or no exposition to natalizumab treatment (Kruskal–Wallis test, for CD4 CM: *p*≤ 0.0001, CD4 EM: *p* = 0.0022, and CD4 E: *p* < 0.0001; for CD8 CM: *p* < 0.0001, CD8 EM: *p* < 0.0001, CD8 E: *p* < 0.0001, and CD8 I: *p* < 0.0001) (Figure 2) (Supplementary Table 2).

**Figure 3:**
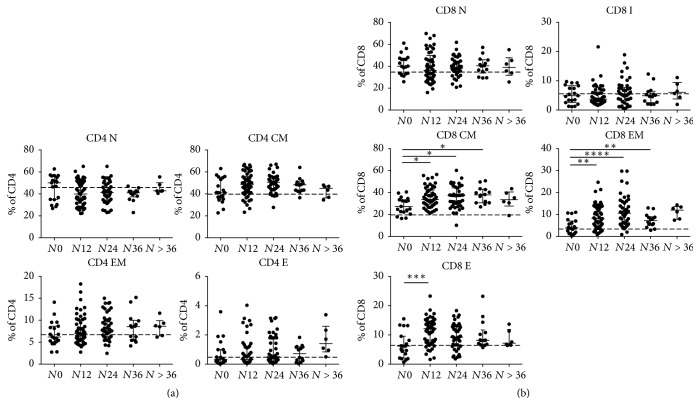
*Evaluation of T-lymphocyte subset percentages in blood samples*. Percentages of CD4+ (a) and CD8+ (b) T-lymphocyte subpopulations in peripheral blood of RRMS patients under natalizumab treatment are represented, after stratification according to natalizumab infusion number. Values are represented as percentage of the parental population (overall CD4 and CD8, resp.). Statistical analysis was performed using a one-way ANOVA test for nonparametrical data (Kruskal–Wallis, for CD4 N, *p* > 0.05; CD4 CM, *p* > 0.05; CD4 EM, *p* > 0.05; CD4 E, *p* > 0.05; CD8 N, *p* > 0.05; CD8 I, *p* > 0,05; CD8 CM, *p* = 0.02; CD8 EM, *p* = 0.0003; CD8 E, *p* < 0.0057). Dunnett's posttest was performed comparing each group with *N*0, and asterisks represent the level of statistical significance. Lines and whiskers represent median values and interquartile ranges, respectively. Dashed line represents median percentages observed in healthy donors. N: naïve, CM: central memory, EM: effector memory, E: effectors, and I: intermediate. *N*0: no infusions, *N*12: from 1 to 12 infusions, *N*24: from 13 to 24 infusions, *N*36: from 25 to 36 infusions, and *N* > 36: over 36 infusions of natalizumab. ^*∗*^0.05 < *p* < 0.01; ^*∗∗*^0.01 < *p* < 0.001; ^*∗∗∗*^*p* < 0.001; ^*∗∗∗∗*^*p* < 0.0001.

**Figure 4:**
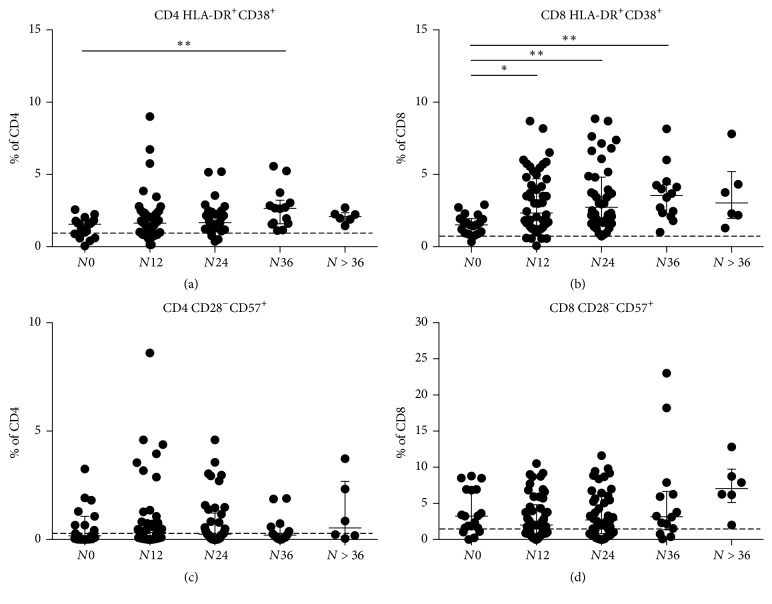
*Immune activation and immune senescence levels of CD4+ and CD8+ T-lymphocytes*. (a) CD4+ T-lymphocyte immune activation levels were assessed considering the percentages of HLA-DR and CD38 double positive CD4. *N*0: 1.6%  [0.9–1.8], *N*12: 1.6%  [0.9–2.3], *N*24: 1.7%  [1.3–2.2], *N*36: 2.7%  [1.6–3.2], and *N* > 36: 2.1  [1.8–2.4]. Statistical analysis was performed using a one-way ANOVA test for nonparametrical data (Kruskal–Wallis, *p* = 0.0099). Dunnett's posttest was performed comparing each group with *N*0, and asterisks represent the level of statistical significance. Dashed line represents median value of CD4+ T-lymphocyte immune activation levels observed in healthy donors. (b) CD8+ T-lymphocyte immune activation levels were assessed considering the percentages of HLA-DR and CD38 double positive CD8. *N*0: 1.5%  [1.0–1.9], *N*12: 2.3%  [1.4–4.7], *N*24: 2.7%  [1.7–4.8], *N*36: 3.6%  [2.3–4.3], and *N* > 36: 3.0%  [2.0–5.2]. Statistical analysis was performed using a one-way ANOVA test for nonparametrical data (Kruskal–Wallis, *p* = 0.0025). Dunnett's posttest was performed comparing each group with *N*0, and asterisks represent the level of statistical significance. Dashed line represents median value of CD8+ T-lymphocyte immune activation levels observed in healthy donors. Immune senescence levels were evaluated for CD4+ (c) and CD8+. (d) T-lymphocytes as the percentages of CD28 negative and CD57 positive cells. No statistical significant differences were found after performing a one-way ANOVA test for nonparametrical data (Kruskal–Wallis). Lines and whiskers represent median values and interquartile ranges, respectively. *N*0: no infusions, *N*12: from 1 to 12 infusions, *N*24: from 13 to 24 infusions, *N*36: from 25 to 36 infusions, and *N* > 36: over 36 infusions of natalizumab. ^*∗*^0.05 < *p* < 0.01; ^*∗∗*^0.01 < *p* < 0.001; ^*∗∗∗*^*p* < 0.001; ^*∗∗∗∗*^*p* < 0.0001.

Analyzing CD8 subsets, we showed that CD8 N percentages were unchanged in the 5 groups, with median values comparable with those observed in HD. Conversely CD8 CM, EM, and E percentages increased in patients with longer exposition compared to patients with shorter or no exposition to natalizumab treatment (Kruskal–Wallis test, for CD8 CM: *p* = 0.02, CD8 EM: *p* = 0.0003, and CD8 E: *p* = 0.0057) (Figure 3(b)). No statistically significant differences in CD4 subset percentages were observed in the 5 groups (Figure 3(a)) (Supplementary Table 2). The assessment of T-lymphocyte immune activation demonstrated an increment of immune activated CD4+ and CD8+ cells in patients with longer exposition compared to patients with shorter or no exposition to natalizumab treatment (Kruskal–Wallis test, *p* = 0.01 and *p* = 0.003, resp.) (Figures 4(a) and 4(b)). No statistically significant differences were found in immune senescent CD4 and CD8 percentages (Figures 4(c) and 4(d)).

### 3.4. Relationship between CD8 E and JCV Replication

**Figure 5:**
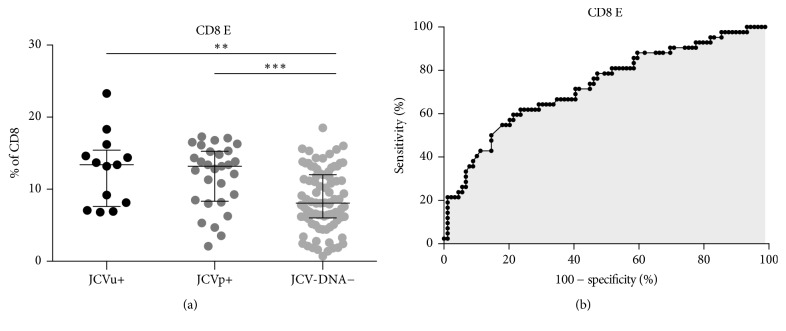
*CD8 E percentages according to JCV-DNA detection in biological samples and ROC analysis*. (a) CD8 E percentages associated with JCV-DNA positive urine samples (JCVu+), JCV-DNA positive plasma samples (JCVp+), and JCV-DNA negative plasma and urine samples (JCV-DNA−) are shown: for JCVu+, 13.4%  [7.6–15.4], for JCVp+, 13.2%  [8.3–15.2], and for JCV-DNA−, 8.0%  [6.0–12.0]. Data are represented as median [interquartile range]. Statistical analysis was performed using a one-way ANOVA test for nonparametrical data (Kruskal–Wallis, *p* = 0.0001). Dunnett's posttest was performed comparing each group with JCV-DNA− group, and asterisks represent the level of statistical significance. (b) ROC analysis was performed using CD8 E percentages after stratification of samples according to JCV detection in plasma and/or urine in JCV-DNA+ and JCV-DNA−. The area under the curve is 0.73 with *p* < 0.0001. The cut-off of >10.6% shows a sensitivity of 67% (CI: 51% to 80%) and a specificity of 65% (CI: 54% to 75%). CI: confidence interval. Lines and whiskers represent median values and interquartile ranges, respectively. CD8 E: CD8 effectors. ^*∗*^0.05 < *p* < 0.01; ^*∗∗*^0.01 < *p* < 0.001; ^*∗∗∗*^*p* < 0.001; ^*∗∗∗∗*^*p* < 0.0001.

Considering that urine and blood were collected together for each patient at each sampling time, 131 coupled samples were obtained from 44 patients. Twenty-nine coupled samples were JCV-DNA+ because of JCV-DNA detection in plasma (JCVp+) while 13 coupled samples were JCV-DNA+ because of JCV-DNA detection only in urine (JCVu+). Eighty-nine coupled samples were JCV-DNA− because of JCV-DNA undetectability in both plasma and urine. After comparing the JCVp+, JCVu+, and JCV-DNA− groups, the percentage of CD8 E was increased in the JCVp+ and JCVu+ compared to the JCV-DNA− group and the differences were statistically significant (Kruskal–Wallis test, *p* = 0.0001) (Figure 5(a)).

Given the evidence of increased CD8 E percentages associated with JCV-DNA positivity in plasma and urine, the Receiving Operator Curve (ROC) was calculated and a cut-off of 10.6% for CD8 E was found, indicating that CD8 E percentages greater than 10.6% were predictive of JCV-DNA detection in either plasma, urine, or both samples (Figure 5(b)).

**Figure 6:**
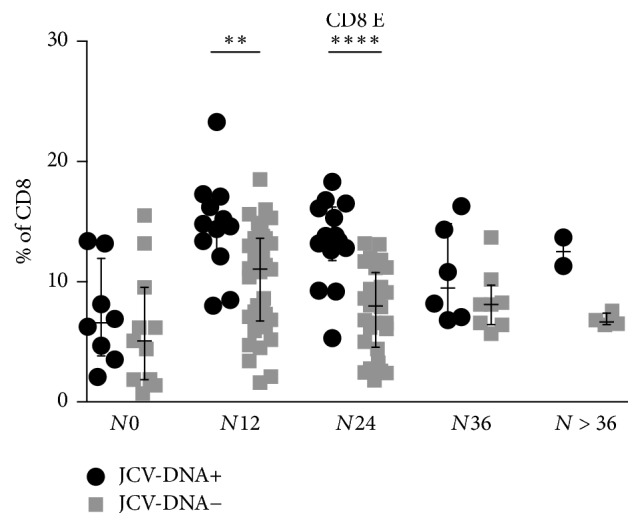
*CD8 E percentages in JCV-DNA+ and JCV-DNA*−* samples, after stratification according to natalizumab infusion number*. Comparison of CD8 E percentages between JCV-DNA+ (plasma and/or urine) and JCV-DNA− samples for *N*0: 6.6%  [3.8–11.9] versus 5.1%  [1.9–9.5], *N*12: 14.7%  [12.4–16.9] versus 11.1%  [6.8–13.6], *N*24: 13.6%  [11.8–16.2] versus 8.0%  [4.6–10.8], *N*36: 9.5%  [7.0–14.8] versus 8.1%  [6.5–9.7], and *N* > 36: 12.5%  [11.3–13.7] versus 6.7%  [6.4–7.4]. Data are shown as median [interquartile range]. Statistical analysis was performed using the 2-tailed Mann–Whitney test (*p* = 0.3, *p* = 0.002, *p* < 0.0001, *p* = 0.2, and *p* = 0.1, for *N*0, *N*12, *N*24, *N*36, and *N* > 36, resp.). Lines and whiskers represent median values and interquartile ranges, respectively. CD8 E: CD8 effectors. *N*0: no infusions, *N*12: from 1 to 12 infusions, *N*24: from 13 to 24 infusions, *N*36: from 25 to 36 infusions, and *N* > 36: over 36 infusions of natalizumab. ^*∗*^0.05 < *p* < 0.01; ^*∗∗*^0.01 < *p* < 0.001; ^*∗∗∗*^*p* < 0.001; ^*∗∗∗∗*^*p* < 0.0001.

After stratifying the samples according to natalizumab infusion number, higher levels of CD8 E were associated with JCV-DNA detection in plasma or urine, for *N*12 (Mann–Whitney, *p* = 0.002) and *N*24 groups (Mann–Whitney, *p* < 0.0001). Although similar results were obtained for *N*36 and *N* > 36 groups, the differences did not reach the statistical significance (Figure 6).

**Figure 7:**
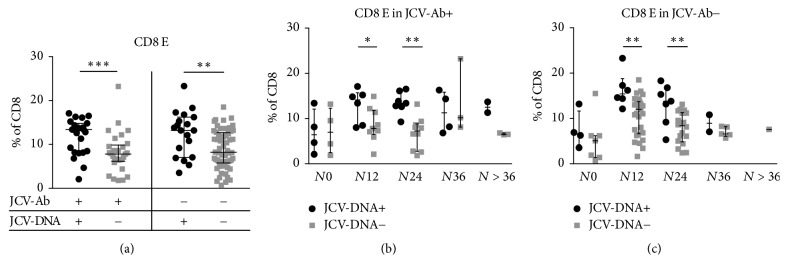
*CD8 E percentages in JCV-DNA+ and JCV-DNA*−* samples, after stratification according to anti-JCV specific serology and natalizumab infusion number*. (a) Comparison of CD8 E percentages between JCV-DNA+ (plasma and/or urine) and JCV-DNA− samples in patients with either positive or negative JCV serology (JCV-Ab+ and JCV-Ab−, resp.). For JCV-Ab+, 13.4%  [8.2–14.8] versus 7.8%  [6.2–9.9]; for JCV-Ab−, 13.2%  [7.1–16.2] versus 8.2%  [5.8–12.7], respectively. Data are shown as median [interquartile range]. Statistical analysis was performed using the 2-tailed Mann–Whitney test (*p* = 0.0007 and *p* = 0.004 for JCV-Ab+ and JCV-Ab− groups, resp.). (b) Comparison of CD8 E percentages between JCV-DNA+ (plasma and/or urine) and JCV-DNA− samples in patients with positive JCV serology (JCV-Ab+), after stratification according to natalizumab infusion number: for *N*0: 6.4%  [2.8–12.1] versus 7.0%  [2.5–12.3], *N*12: 14.1%  [8.4–15.7] versus 7.8%  [6.5–11.8], *N*24: 13.4%  [12.6–16.1] versus 7.3%  [2.8–8.8], *N*36: 11.3%  [7.2–15.8] versus 10.2%  [8.1–23.2], and *N* > 36: 12.5%  [11.3–13.7] versus 6.5%  [6.4–6.8]. Data are shown as median [interquartile range]. Statistical analysis was performed using the 2-tailed Mann–Whitney test (*p* = 0.8, *p* = 0.049, *p* < 0.0012, *p* = 0.86, and *p* = 0.2, for *N*0, *N*12, *N*24, *N*36, and *N* > 36, resp.). (c) Comparison of CD8 E percentages between JCV-DNA+ (plasma and/or urine) and JCV-DNA− samples in patients with negative JCV serology (JCV-Ab−), after stratification according to natalizumab infusion number: for *N*0: 6.6%  [4.2–11.6] versus 5.1%  [1.4–6.2], *N*12: 15.4%  [13.8–18.8] versus 12.0%  [6.7–13.7], *N*24: 13.8%  [9.2–16.8] versus 8.4%  [4.9–11.3], *N*36: 8.9%  [7.1–10.8] versus 6.6%  [6.1–8.2], and *N* > 36: not applicable. Data are shown as median [interquartile range]. Statistical analysis was performed using the 2-tailed Mann–Whitney test (*p* = 0.2, *p* = 0.004, *p* < 0.008, *p* = 0.38, and *p* = not applicable, for *N*0, *N*12, *N*24, *N*36, and *N* > 36, resp.). Lines and whiskers represent median values and interquartile ranges, respectively. CD8 E: CD8 effectors. *N*0: no infusions, *N*12: from 1 to 12 infusions, *N*24: from 13 to 24 infusions, *N*36: from 25 to 36 infusions, and *N* > 36: over 36 infusions of natalizumab. ^*∗*^0.05 < *p* < 0.01; ^*∗∗*^0.01 < *p* < 0.001; ^*∗∗∗*^*p* < 0.001; ^*∗∗∗∗*^*p* < 0.0001.

These data were further analyzed, considering JCV serostatus and stratifying samples according to JCV serology in JCV-Ab+ and JCV-Ab−. Notably, the results previously shown for the whole cohort were confirmed for both groups of JCV-Ab+ and JCV-Ab− patients (Mann–Whitney, *p* < 0.001 and *p* = 0.004, resp.) (Figure 7(a)). Interestingly, CD8 E percentages were found significantly increased in JCV-Ab+ as well as JCV-Ab− patients with detectable JCV-DNA in urine or plasma, for *N*12 and *N*24 groups (Mann–Whitney, *p* = 0.049 and *p* = 0.0012 for JCV-Ab+; *p* = 0.004 and *p* = 0.008 for JCV-Ab−, resp.) (Figures 7(b) and 7(c)).

## 4. Discussion

Our results confirmed the presence of a subset of patients with negative JCV serology (indicating the lack of anti-JCV specific IgG) despite the detection of viral DNA in blood or urine. Other authors reported similar results, suggesting that JCV serology alone is not sufficient for the diagnosis of JCV infection and should be associated with JCV-DNA detection in body fluids (such as blood or urine) in order to truly identify JCV infected patients [25, 26]. Possible explanations for this discordance could be the occurrence of acute infection at sampling time, when IgG are still absent despite a replicating virus, or the presence of a low IgG titer, not detectable by the Stratify JCV assay [25]. Second-generation Stratify JCV assay showed an increased sensitivity, detecting anti-JCV specific antibodies in previously negative patients [27]. Despite this improvement, some patients still have a detectable JCV-DNA in urine or blood with a negative JCV serology. Some authors described the occurrence of PML in RRMS patients receiving natalizumab with a negative JCV serology [28], thus underlining the need for a more accurate evaluation of those patients who should start or are already receiving a treatment with natalizumab, by using different methods to directly or indirectly detect prior JCV infection. In this study we observed that CD49d expression tends to decrease on T-lymphocytes in parallel with the increase in natalizumab infusion number. This result was already reported in previous works from our and other groups [17, 29]. Notably, in this work we adopted a cross-sectional approach, demonstrating that CD49d expression can be used as a marker of natalizumab exposure. Furthermore, the decrease in CD49d expression on T-lymphocytes was inversely related to natalizumab infusion number, following a linear trend. CD49d expression has been already proposed as a useful tool for monitoring natalizumab efficacy, especially in those patients who become refractory to treatment, because of the development of anti-natalizumab antibodies. Indeed, anti-natalizumab antibody production was associated with an increase in CD49d expression after an initial decrement [29]. Recently, the evaluation of CD49d receptor occupancy with flow cytometry has been considered as a method to optimize and personalize natalizumab treatment in RRMS patients [30]. As a consequence of the decreased CD49d expression on the cell surface, we observed a modification of CD8 subset distribution, with accumulation of CD8 central memory, effector memory, and effector subsets in peripheral blood. Similar results have been previously shown with a longitudinal approach in natalizumab treated RRMS patients [17, 22]. Furthermore, CD4/CD8 ratio was found decreased in patients under natalizumab treatment with a negative correlation with JCV antibody index [22]. Planas et al. showed an increase in peripheral blood lymphoid progenitor percentages and B- and T-lymphocyte absolute counts of RRMS patients after natalizumab treatment, although they did not find any difference in CD4 and CD8 subset distribution and CD4/CD8 ratios. These differences could be due to the heterogeneity of the cohorts, considering that, for instance, in our study, patients were younger and had lower EDSS values compared to those enrolled in Planas et al. study [31]. In a previous work in which 26 RRMS patients under natalizumab treatment were longitudinally followed up for 24 months, we showed that CD8 E is increased in patients with detectable JCV-DNA in plasma or urine [17]. The present work corroborates our previous results, demonstrating similar findings in a larger cohort of patients and adopting a cross-sectional approach for data analysis. CD8 E tended to increase in RRMS patients treated with natalizumab and also increased in patients with JCV-DNA detection in plasma or urine. This finding indicates a potential role of these cells during JCV reactivation. Memory and effector CD8 subset accumulation in peripheral blood, due to natalizumab administration, should reflect their reduction in the CNS [32], with an impairment of immune-surveillance mechanisms. It has been shown that low CD8 counts may increase the risk of PML, because JCV containment appears to be more strongly related to CD8+ T-lymphocyte activity [33]. Moreover, dimethyl fumarate (another effective treatment for RRMS) causes significantly sustained reduction in CD8+ T-lymphocyte counts and, to a lesser extent, CD4+ T-lymphocyte counts [34, 35]. Gieselbach et al. showed that in 19 PML cases observed in patients treated with fumaric acid esters, low CD8 and CD4 cell counts were associated with increased risk of developing PML [36]. Although we did not observe any case of natalizumab-induced PML in this cohort of patients, we analyzed the association between CD8 subset modifications and JCV reactivation. Here we confirmed previous results [17], demonstrating that CD8 E percentages were increased in patients with detectable JCV-DNA in urine and plasma. Moreover, we found a cut-off value for CD8 E percentages of 10.6%, which means that CD8 E percentages above this value were strongly associated with JCV-DNA detection. After stratifications of RRMS patients according to natalizumab infusion number, we demonstrated that the combined effect of natalizumab and JCV replication determined an increase in CD8 E percentages, with statistically significant differences in the *N*12 and *N*24 groups, while no differences were observed for *N*0 group (before natalizumab administration). Finally, after stratifying the patients according to JCV serology, CD8 E percentages were found to be increased, in association with JCV-DNA detection in urine or plasma, in patients with JCV positive as well as negative serology, indicating that CD8 E percentages increase in parallel with JCV-DNA detection, independently from anti-JCV serostatus. Some limitations of our study were the fact that we did not observe any case of natalizumab-induced PML and that we did not evaluate the specificity of CD8 subsets, which accumulate in peripheral blood after natalizumab treatment. Functional studies involving peripheral blood CD8 subsets, after stimulation with JCV specific peptides, could give more information about their specificity and the ability to produce cytokines and perform cytotoxicity.

## 5. Conclusion

The most important finding of this study is that, in natalizumab treated RRMS patients, independently from JCV serology, CD8 E percentages were found increased in those subjects with JCV replication, as assessed by viral DNA detection in plasma or urine samples. Moreover, the evaluation of CD8 E could help in detecting JCV reactivation, especially in JCV seronegative patients, who are currently considered at lower risk of developing PML during natalizumab treatment. Larger cohorts are needed in order to verify these results and further studies should be performed in order to understand the pathological and clinical significance of CD8 subset modifications associated with natalizumab administration and their relationship with JCV reactivation and PML onset.

## Figures and Tables

**Table 1:** Demographic and clinical features of HD and RRMS patients.

	HD	RRMS patients				
		*N*0	*N*12	*N*24	*N*36	*N* > 36
*N*	15	19	29	32	12	6
F/M	7/8	7/12	13/17	17/14	8/4	2/4
Median age in years [IQR]	30 [27–35]	29.5 [26–38]	46.5 [37–50.3]	40 [38–42.5]	38 [29–40]	40.5 [33.5–47.5]
Median years of disease [IQR]	-	7.5 [5.2–9.7]	11.5 [9.2–18.7]	14 [8–23]	9 [7.25–11.7]	7 [6–9.5]
Median EDSS^*∗*^ [IQR]	-	2 [0.5–2]	1 [1–1.5]	2.5 [2.1–2.8]	1 [1-2]	1 [1–1.75]
No therapy^*∗∗*^ (/N)	-	4/19	6/29	8/32	4/12	4/6
Interferon *β*^*∗∗*^ (/N)	-	13/19	20/29	19/32	7/12	2/6
Mitoxantrone and interferon *β*^*∗∗*^ (/N)	-	1/19	1/29	1/32	0/12	0/6
Glatiramer acetate^*∗∗*^ (/N)	-	1/19	2/29	4/32	1/12	0/6

HD: healthy donors, *N*0: 0 infusions, *N*12: from 1 to 12 infusions, *N*24: from 13 to 24 infusions, *N*36: from 25 to 36 infusions, and *N* > 36: over 36 infusions of natalizumab. *N*: total number of patients; F: female; M: male; IQR: interquartile range;  ^*∗*^EDSS: Expanded Disability Status Scale, with values ranging from 0 (normal neurological examination) to 10 (bedridden patient) (1);  ^*∗∗*^therapy before starting natalizumab.

## Supplementary Materials

Supplementary Materials Supplementary Table 1: anti-JCV serostatus assessed with the Stratify JCV assay. Supplementary Table 2: CD49d median fluorescence intensity (MFI) and percentages of peripheral blood CD4+ and CD8+ T-lymphocyte subsets.
