# Supplementary material for: JC Virus-DNA Detection Is Associated with CD8 Effector Accumulation in Peripheral Blood of Patients with Multiple Sclerosis under Natalizumab Treatment, Independently from JC Virus Serostatus
Source: Biomed Res Int. 2018 Feb 27;2018:5297980. doi: 10.1155/2018/5297980 (PMC5848061; doi:10.1155/2018/5297980)
Supplement: Supplementary Materials — Supplementary Table 1: anti-JCV serostatus assessed with the Stratify JCV assay. Supplementary Table 2: CD49d median fluorescence intensity (MFI) and percentages of peripheral blood CD4+ and CD8+ T-lymphocyte subsets. [file 5297980.f1.docx]

**Supplementary table 1: Anti-JCV serostatus assessed with the Stratify JCV assay**

| **Group** | **T0** | **T12** | **T24** | **T36** | **T>36** |
| --- | --- | --- | --- | --- | --- |
| **N of serum samples** | 19 | 50 | 42 | 14 | 6 |
| **Stratify JCV negative N** | 11 | 35 | 25 | 7 | 1 |
| **Stratify JCV I positive N** | 6 | 5 | 0 | 3 | 0 |
| **Stratify JCV II positive N median index [IQR]** | 2 1,31 [0,82-1,81] | 10 0,98 [0,33-3,9] | 17 0,87 [0,57-1,59] | 4 2,14 [0,62-3,82] | 5 0,52 [0,50-0,66] |

N: number; Stratify JCV I: first-generation Stratify JCV assay (index values for positive samples not available); Stratify JCV II: second-generation Stratify JCV assay (index values for positive samples available); IQR: interquartile range.

**Supplementary table 2: CD49d Median Fluorescence Intensity (MFI) and percentages of peripheral blood CD4+ and CD8+ T-lymphocyte subsets.**

|  |  | HD | RRMS | | | | |
| --- | --- | --- | --- | --- | --- | --- | --- |
|  | Cell type |  | **N0** | **N12** | **N24** | **N36** | **N>36** |
| CD49d MFI |  |  |  |  |  |  |  |
|  | CD4 | 464 [438-516] | 462.5 [400-464] | 411.5 [269.5-505.8] | 258 [183-365] | 207 [177.5-409] | 292 [207-333] |
|  | CD4 N | 295 [281.8-301.8] | 338 [155.3-412.5] | 362 [137-518.5] | 193 [148.5-374] | 273 [220-297] | 323 [313.5-331.5] |
|  | CD4 CM | 769.5 [703.8-920.5] | 693 [526.5-941.8] | 523.5 [408-663.8] | 287 [192-500.5] | 350 [219-433.5] | 196 [166.6-321] |
|  | CD4 EM | 961 [827-1095] | 944 [611.5-1218] | 527 [409.5-629.3] | 369 [212-794] | 480 [360-762.5] | 245.5 [181.5-493.8] |
|  | CD4 E | 1061 [431-1089] | 722 [631.8-1281] | 361.5 [323.3-446.3] | 453 [329-819] | 368 [150-422.5] | 245.5 [214-370] |
|  | CD8 | 1011 [815-1060.5] | 655 [671-1096] | 491.5 [259.3-659] | 427.5 [203-767.5] | 296 [195.5-472] | 453 [340.8-468.8] |
|  | CD8 N | 572.5 [531.8-604.3] | 459.5 [311.8-593.3] | 459.5 [245-705] | 413 [242-559.5] | 390 [225.5-602] | 442.5 [269.5-529.8] |
|  | CD8 CM | 1054 [991-1152] | 895 [565-1071] | 474 [363-613.5] | 435 [308-532] | 340 [244.5-459.5] | 362 [240.3-409.5] |
|  | CD8 EM | 1167 [1124-1220] | 983 [752-1155] | 707.5 [566-825] | 562 [399.5-758.5] | 471 [316.5-666.5] | 300 [243.3-344.8] |
|  | CD8 I | 1031 [963.3-1079] | 841 [601-1031] | 700.5 [580-972.5] | 528 [462-731] | 430 [260.5-531] | 293.5 [215.8-360.8] |
|  | CD8 E | 1057 [1020-1109] | 992 [613-1167] | 756 [654.3-994.5] | 481 [306-614] | 359 [210-492] | 333 [309.5-409.5] |
| Percentages |  |  |  |  |  |  |  |
|  | CD4 | 50.8 [41.6-62.8] | 52.4 [33.6-57.3] | 52.9 [43.7-59.3] | 57.7 [48.5-61.8] | 57.5 [52.7-62.5] | 56.8 [54-62.5] |
|  | CD4 N | 47.3 [44.7-59.3] | 50.1 [34.8-56.4] | 39.9 [31.5-51.1] | 41.2 [34.5-49.7] | 41 [37.2-42.4] | 42.8 [42-50.1] |
|  | CD4 CM | 40.4 [36.6-42.9] | 41.1 [35.3-54.8] | 48.6 [40.9-55.8] | 50.6 [44.6-55.1] | 48.2 [43.1-51] | 45.1 [36.3-47.1] |
|  | CD4 EM | 5.9 [5.3-9.9] | 6.1 [5.1-8.6] | 6.9 [5.5-10.1] | 7.6 [6.1-10.3] | 8.5 [5.4-9.9] | 8.6 [6.4-9.8] |
|  | CD4 E | 0.6 [0.5-1.0] | 0.3 [0.1-0.9] | 0.3 [0.1-1.1] | 0.5 [0.2-1.8] | 0.7 [0.2-1.1] | 1.4 [0.8-2.5] |
|  | CD8 | 25 [24.5-25.5] | 27.8 [23.9-32.5] | 31.7 [25.7-35.1] | 31.9 [28.9-34.7] | 32.9 [28-36.8] | 38.8 [36.1-41] |
|  | CD8 N | 38.1 [26.9-43.6] | 40.1 [33.6-47.3] | 36.3 [29-49.8] | 38.1 [35-44.7] | 40.4 [33.8-45.9] | 38.9 [31.8-47.7] |
|  | CD8 CM | 20.3 [15.8-23.6] | 27.4 [22.2-33.4] | 33.7 [29.5-42.3] | 37.4 [29.1-44.3] | 37.9 [30.6-42.9] | 33.5 [27.7-40.6] |
|  | CD8 EM | 4.6 [2.2-5.6] | 3.9 [1.3-7] | 8.2 [4.5-13.6] | 10.5 [5.6-15.9] | 7.1 [5-8.9] | 11.9 [7.8-13.6] |
|  | CD8 I | 6.0 [3.6-8.8] | 4.9 [2.5-8.3] | 3.7 [2.7-6.1] | 5.4 [3.3-7.7] | 4.9 [2.5-6.4] | 6 [3.7-9.4] |
|  | CD8 E | 7.0 [6.1-16] | 6.2 [2.1-9.5] | 12.1 [7.4-14.4] | 9.2 [6.1-13.1] | 8.1 [6.7-11.6] | 7.1 [6.4-11.9] |

Data are shown as median [interquartile range].

RRMS: relapsing-remitting multiple sclerosis; HD: healthy donors; N: naïve, CM: central memory, EM: effector memory, E: effectors, I: intermediate. N0: no infusions, N12: from 1 to12 infusions, N24: from 13 to 24 infusions, N36: from 25 to 36 infusions, N>36: over 36 infusions of natalizumab.
